# Why conferences matter – musings from a crystallographic meeting

**DOI:** 10.1107/S2052252519002215

**Published:** 2019-02-21

**Authors:** Edward N. Baker

**Affiliations:** a School of Biological Sciences, University of Auckland, Auckland, New Zealand

**Keywords:** conferences, crystallographic meetings, cryo-EM, microED, XFEL

## Abstract

Despite extraordinary advances in the options available for instant electronic communication, the argument is made that conferences still provide a unique forum for transmission of new ideas and advances in science, with their ability to inspire attendees.

At a time when we enjoy instant communication and unprecedented access to information *via* the internet, it is easy to question some of the traditional ways in which advances in science are communicated. Printed journals have now largely disappeared from most peoples’ work places, while at the same time more journals than ever before are now available electronically. In this environment one might ask (as I have heard colleagues ask from time to time): ‘Why should I travel all this distance, and take so much time out of my work, just to attend this or that conference?’

We have recently hosted, in Auckland, the 15th Conference of the Asian Crystallographic Association (AsCA), jointly with the annual meeting of the Society of Crystallographers in Australia and New Zealand. As anyone who has organized a similar conference will know, this was a lot of work. We worried about whether enough people would come, given the distance, about facilities and sponsors, and about gender and age balance, geographical balance and choice of topics. In the event, however, it was immensely exciting and stimulating, exceeding all our expectations. We had nearly 500 participants – crystallography is growing fast in Asia – and as I sat listening to some of the great speakers, I got to thinking about how valuable such meetings are. Hence this editorial.

Throughout my career, I have been inspired by things I have heard at crystallographic conferences. Sometimes it has been a new method, or a new twist on an old method. Or it may be a neat practical solution to some problem a speaker has been confronted with, that makes me think afresh about something I or one of my colleagues have struggled with. Or it may simply be a boost to one’s confidence: ‘If they can do that, why can’t we?’ And there is the opportunity to talk to people in the all-important coffee breaks and lunch periods, or to seed future collaborations.

What first attracted me to crystallography was its unique blend of disciplines: chemistry, physics and biology, all underpinned in a rigorous way by mathematics. It is this diversity that is one of the great strengths of crystallographic meetings, enabling new technological advances to be rapidly taken up by different areas of science. It was also a major factor that led to the establishment of this journal, with its goal of presenting the applications of crystallography and related techniques across the full spectrum of the natural sciences; a mirror for the excitement of crystallographic meetings.

The emergence of new technologies is particularly striking in biology today. For me, the highlights of the 2017 IUCr Congress in Hyderabad were the sessions on the spectacular advances of cryo-electron microscopy (cryo-EM), which make it both a competitor and a complement to X-ray crystallography as a tool for biological structure determination. There are so many common elements to these two approaches, including sample preparation, model building into maps and validation, that researchers can move easily between one and the other according to the dictates of a particular problem. Another example is in the applications of X-ray free electron laser (XFEL) technologies, which have in turn stimulated the development of serial crystallography approaches at new-generation synchrotrons. At last we have new and powerful ways to approach dynamic processes (Spence, 2018 ▸). Developments in XFEL and serial crystallography have found a natural home in this journal, like those in cryo-EM. All of them were highlighted at our recent AsCA meeting, as they also are today at other crystallographic meetings round the world.

My personal highlight from our Auckland meeting, however, was the demonstration by Tamir Gonen of the enormous potential of another variant of the crystallographic method, microelectron diffraction (microED). Electron diffraction has been used for many years, mostly in materials science. The innovative idea in more recent applications of microED, however, is the use of cryo-EM to select out individual microcrystals too small for X-ray diffraction and solve their structure by electron crystallography (Sawaya *et al.*, 2016 ▸; Rodriguez *et al.*, 2017 ▸). After overcoming some methodological problems, it has now become apparent that far from being a ‘fringe’ technique, applicable to a few special cases, microED provides a rapid and powerful way to obtain high resolution structures for both macromolecular and small-molecule samples – with the ability even to analyse microcrystalline mixed powders of chemical compounds (Jones *et al.* (2018 ▸). I sat in the audience, thinking in awe about all the new opportunities that are now presenting themselves – see, for example, Lanza *et al.* (2019 ▸) and Zee *et al.* (2019 ▸) in this issue of **IUCrJ**.

It is possible that some other forms of instant communication can capture the immediacy and excitement of such work, but I am all the more convinced of the value of the direct, personal communication that is experienced in scientific meetings.

## References

[bb1] Jones, C. G., Martynowycz, M. W., Hattne, J., Fulton, T., Stoltz, B. M., Rodriguez, J. A., Nelson, H. M. & Gonen, T. (2018). *ACS Cent. Sci.* **4**, 1587–1592. 10.1021/acscentsci.8b00760 PMC6276044 30555912

[bb2] Lanza, A., Margheritis, E., Mugnaioli, E., Cappello, V., Garau, G. & Gemmi, M. (2019). *IUCrJ*, **6**, 178–188. 10.1107/S2052252518017657 PMC6400191 30867915

[bb3] Rodriguez, J. A., Eisenberg, D. S. & Gonen, T. (2017). *Curr. Opin. Struct. Biol.* **46**, 79–86. 10.1016/j.sbi.2017.06.004 PMC5683914 28648726

[bb4] Sawaya, M. R., Rodriguez, J., Cascio, D., Collazo, M. J., Shi, D., Reyes, F. E., Hattne, J., Gonen, T. & Eisenberg, D. S. (2016). *Proc. Natl Acad. Sci. USA*, **113**, 11232–11236. 10.1073/pnas.1606287113 PMC5056061 27647903

[bb5] Spence, J. C. H. (2018). *IUCrJ*, **5**, 236–237. 10.1107/S2052252518005365 PMC5929370 29755740

[bb6] Zee, C.-T., Glynn, C., Gallagher-Jones, M., Miao, J., Santiago, C. G., Cascio, D., Gonen, T., Sawaya, M. R. & Rodriguez, J. A. (2019). *IUCrJ*, **6**, 197–205. 10.1107/S2052252518017621 PMC6400192 30867917

